# Long-term changes of alpine stream macroinvertebrates in relation to glacial recession

**DOI:** 10.1007/s00027-025-01232-9

**Published:** 2025-10-03

**Authors:** S. Bruni, L. Wanner, A. Grolimund, C. T. Robinson

**Affiliations:** 1 Department of Aquatic Ecology, Eawag, 8600 Duebendorf, Switzerland; 2 Institute of Integrated Biology, ETH-Zürich, 8092 Zurich, Switzerland

**Keywords:** Lake outlet, Aquatic insects, Spatio-temporal, Colonization, Temperature

## Abstract

Alpine landscapes are being rapidly transformed, being fueled by ongoing environmental change and glacial recession. Glacial recession has caused emergence of new stream channels as well as changes in the physico-chemistry of surface waters embedded in glaciated landscapes. Our research examined temporal changes in the physico-chemistry and macroinvertebrates during (1) the elongation of a glacial stream and (2) among different alpine lake networks (inlets, outlets, and downstream sites) over the last 24/25 years. The glacial stream study revealed rapid colonization of the emergent stream following deglaciation as well as colonization of previous sites by novel taxa (some from lower elevations). The water physico-chemical data revealed changes in the habitat template of sites over the study period, especially in respect to water temperature and turbidity. The lake network study also showed changes in macroinvertebrate assemblages over the observation period as well as differences on the basis of lake location and type. Here, lake outlet water temperatures increased more over time at northern than southern Alpine lakes. Further, kryal and rhithral lake outlets differed in response regarding water physico-chemistry and macroinvertebrate diversity. Both studies highlight the importance of monitoring alpine surface waters for better understanding of abiotic and biotic responses to landscape transformation resulting from ongoing and rapid environmental change, especially in relation to glacial recession.

## Introduction

The hydrographic networks of alpine catchments are complex due to diverse water sources, including groundwater-fed (krenal), precipitation-fed (rhithral), and glacier melt (kryal) waters. These water sources have specific hydrological and physico-chemical conditions that contribute to the high environmental heterogeneity of alpine waters (Ward 1994; Füreder et al. 2001). Within the alpine realm of freshwater ecosystems, a diversity of lakes also play an important role in the ecohydrology of fluvial networks (Robinson et al. 2022). Alpine lakes can act as sinks for particles, including particulate phosphorus, total suspended solids, and atmospheric nitrogen deposition (Hieber et al. 2002), and along with their generally small size, renders them sensitive to environmental and climatic change (Beniston et al. 1997; Veit 2002; Thompson et al. 2005; Catalan et al. 2006). In the context of this study, alpine lake outlets are fluvial systems and show specific attributes due to the lake effect; e.g., water temperature is more stable and overall higher compared with similar alpine streams (Harding 1992; Hieber et al. 2002) and the flow in outlet streams is more moderate and stable, providing distinct habitats for macroinvertebrates (Robinson and Minshall 1990; Milner and Petts 1994). Further, the water source of the lake also influences the habitat template of respective lake outlets. In kryal systems, the lake mitigates flow and temperature fluctuations as well as seasonality of chemical characteristics in a more pronounced manner than in rhithral systems (Milner and Petts 1994; Hieber et al. 2002). On the other hand, rhithral lake outlets typically have higher periphyton production than other alpine rhithral streams (Hieber et al. 2005).

In general, alpine rhithral streams show higher taxa richness than kryal waters (Hieber et al. 2002, 2005). In addition, there seems to be little difference in species composition between kryal streams and kryal lake outlets. Specifically, water temperature significantly influences macroinvertebrate assemblages in glacier-fed waters (Castella et al. 2001). Rhithral lake outlets, on the other hand, stand out and their species composition differs from other rhithral as well as kryal streams (Hieber et al. 2005). Here, noninsect taxa are abundant, including Oligochaeta and nematodes as well as lake-derived copepods and ostracods (Hieber et al. 2005; Čiamporová-Zaťovičová et al. 2010). Lastly, the altitude dependency of aquatic insects makes them more susceptible to climate-induced upslope species range shifts in comparison with noninsect fauna (Čiamporová-Zaťovičová et al. 2010; Bartels et al. 2021).

Glacial runoff maintains the hydrological balance of alpine waters in a relatively deterministic manner (Milner et al. 2017). For instance, in the European Alps, flow magnitudes change over an annual cycle, with maximum flow during warm summer months when ice melt is high and lower flow in cold winter months as glaciers freeze. Glacial melt during warm periods reduces stream intermittency and the loss of surface waters (Slemmons et al. 2013). However, extensive glacial recession poses a threat to the stability of these systems (Milner et al. 2017). For small glaciers such as those found in the Alps, recent models forecast a decrease in runoff in the next decades (Huss et al. 2017). The lower runoff especially affects summer flows, leading to more variable flow magnitudes that are more dependent on precipitation (Milner et al. 2017). Higher water temperatures and increased stream intermittency are possible consequences, and could profoundly affect local flora and fauna (Jacobsen et al. 2014; Siebers et al. 2020; Chanut et al. 2022). Increased glacial ablation is not only changing the flow regime of alpine running waters, but also the chemical composition of streams. For instance, Sommaruga-Wögrath et al. (1997) related changes in the water chemistry of glacial streams with enhanced weathering caused by rising temperatures. Further, kryal systems are generally associated with high turbidity and conductivity originating from high contents of glacial flour and suspended solids (Milner and Petts 1994; Thies et al. 2007; Hood and Berner 2009). These major changes in water chemistry with glacial recession can result in compositional shifts in aquatic communities (Dulić et al. 2014; Pezsek et al. 2022).

During the last few decades, glaciers worldwide have been receding and some have already disappeared. It is estimated that Swiss glaciers lost half their mass between 1931 and 2016 (Mannerfelt et al. 2022), and since the 1970s, an above average over all warming of 1.8 °C has been documented (BAFU 2020). Global warming is especially evident in alpine regions, where organisms are adapted to the relatively cold and harsh conditions of alpine streams (Füreder 1999; Lencioni and Spitale 2015). Small glaciers, in particular, incur the greatest relative mass loss (Huss et al. 2017) such as the Tschierva and Roseg glaciers in the southeast Alps that are part of the present study. Although glacier shrinkage initially augments annual glacier runoff, this flow subsequently diminishes with complete ice disappearance (Milner and Petts 1994; Huss et al. 2008). Few species are found at these high elevations and endemism is prevalent (Muhlfeld et al. 2011). The dramatic loss of permanent ice reshapes the landscape and confronts local biota with novel environments as well as new competitors. These environmental shifts, along with alterations in hydrologic regimes and glacial recession, exert substantial impacts on alpine ecosystems and the kind of habitats available for aquatic organisms over time (Fornaroli et al. 2019; Meißner et al. 2019; Larsen et al. 2021). Further, a warming climate leads to reduced summer precipitation and drier soil conditions, exacerbating the impact of climate change on alpine lakes and associated lake outlets (Thompson et al. 2005). Forecasts indicate that future climate changes will lead to extended ice-free periods and elevated surface-water temperatures in alpine lakes, and thus outlet streams (Gabathuler 1999).

Macroinvertebrates are widely used as indicator organisms in running waters, as they show high sensitivity to environmental conditions (Dou et al. 2022). Physico-chemical properties influence their distributions in a deterministic way (Southwood 1977), and are reflected in the composition of biotic assemblages (Castella et al. 2001; López-López and Sedeño-Díaz 2015). While alpine systems, at high elevation with little human impact, are generally viewed as pristine waters, macroinvertebrates are still useful toward understanding temporal changes in the physical–chemical properties of streams (Bitušík et al. 2010). Ecological thresholds within alpine ecosystems underscore the general decreases in abundances and richness as elevation increases (Füreder et al. 2006; De Mendoza and Catalan 2010). Declining richness and diversity with increasing altitude can indicate a shift from biotic to abiotic drivers in community assembly (Füreder et al. 2000). For instance, macroinvertebrates living near the glacier snout are adapted to the prevailing conditions; they can withstand the near freezing temperatures, high turbidity from glacial flour, and unstable substrata of proglacial waters (Lencioni and Bernabò 2017). Relatively few species can survive in such harsh environments (Jacobsen and Dangles 2012). Here, the dominant taxa is the dipteran subfamily Diamesinae, which are found globally in alpine waters (Ward 1994; Robinson et al. 2016a). As the influence of glacial meltwater declines, dipterans such as Clinocerinae, *Corynoneura* spp., Simuliidae, Ceratopogoninae, and Tanypodinae as well as the mayflies *Rhithrogena* sp. and *Baetis* sp. start to appear (Becquet et al. 2022). In non-glacial alpine streams, chironomids are abundant taxa as well (Alther et al. 2019). As glaciers recede, novel stream habitats emerge and are colonized by macroinvertebrates (Finn et al. 2010). Upward shifts by macroinvertebrates can be expected, as proglacial sites move upstream with the recessing glacier (Hodkinson and Jackson 2005; De Mendoza and Catalan 2010). In terrestrial alpine ecology, a clear upward shift along the temperature gradient has often been shown by biotic assemblages (Beckage et al. 2008; Kerner et al. 2023).

Our research focused on alpine stream macroinvertebrates under two environmental settings: one study examined the longitudinal changes in assemblages along a glacial stream over 24/25 years in relation to glacial recession, whereas the other assessed assemblage changes in alpine lake networks (inlets, outlets, downstream reaches) over the same period. In both cases, we also evaluated temporal changes in physico-chemical properties of the study sites. In the longitudinal study, we expected the habitat template of sites in the earlier years would move upward with glacial recession with the site at the glacial snout displaying typical proglacial stream characteristics. Here, we examined macroinvertebrate assemblage shifts and how they related to longitudinal changes in the physical–chemical template of the proglacial stream. In contrast, the lake network study compared 6 different inlet, outlet, and downstream reaches, providing important data on the sensitivity of alpine lakes to environmental change as reflected in consequent effects on outlet stream assemblages. Our working hypothesis was that assemblages occurring at the different sites in both studies would reflect the respective changes in the physico-chemical habitat template that occurred over the study period. In the longitudinal study, these changes would correspond to the upward shift in habitat properties associated with proglacial streams. In the lake network study, we expected different lake types to show different responses to climate-related environmental change and these changes would be reflected especially in the lake outlet assemblages. Here, lakes were situated in the northern and southern Alps, comprising proglacial lakes (lakes influenced by glacial runoff) and rhithral lakes (lakes not influenced by glacial runoff).

## Study site descriptions

### Longitudinal stream study

**Fig. 1:**
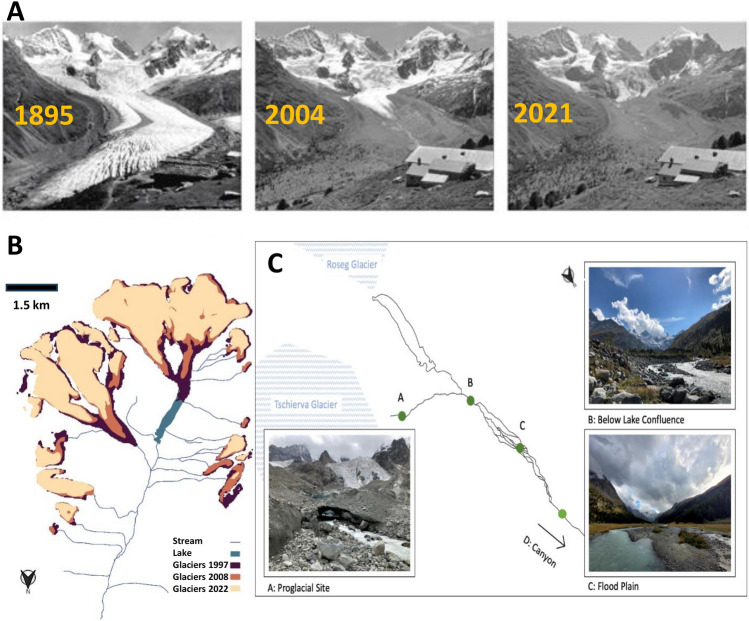
**A** Photographic timeline of glacial recession in the Roseg valley from the years 1895, 2004, and 2021 (photo 1: Schweizerisches Alpines Museum, photo 2 and 3: Jürg Alean). **B** Glacial coverage of the Roseg catchment above the canyon stretch for the different sampling years (1997, 2008, 2022). **C** Positions of the four different catchment zones (A–D) used in comparing the different study years. In the text and figures, Zone A is called glacial snout, Zone B is called lake confluence, Zone C is called flood plain, and Zone D is called canyon

The Roseg valley is in the Bernina Massif in southeast Switzerland (Fig. 1). With its near natural conditions, it is an excellent landscape to investigate the influence of climate change on fluvial waters (Guo et al. 2018). The braided floodplain in the upper valley provides heterogeneous habitats for macroinvertebrates (Iskin and Wohl 2023), a prerequisite for biodiversity (Yachi and Loreau 1999; Tokeshi and Arakaki 2012). The system has been extensively studied over the last 25 years (Ward et al. 1998; Burgherr and Ward 2001; Ward and Uehlinger 2003; Siebers et al. 2020; Chanut et al. 2022), and therefore invaluable in understanding ecological processes from a temporal perspective. The primary water inputs to the mainstream (Roseg river) are from two glaciers located at the head of the valley (Fig. 1B); at the southern end of the valley lies the Roseg glacier, at the southeastern end lies the Tschierva glacier. The moraine of the Tschierva glacier acts as a dam in front of the Roseg glacier behind which a proglacial lake originated around 100+ years ago. The two glaciers used to form one ice mass (Roseg glacier) that ran down the valley, until they retracted and separated in 1934 (Ward and Uehlinger 2003). The glaciated area in the catchment above the canyon amounted to 34% in 1997, 29% in 2008, and 24% in 2021. Runoff of the Tschierva glacier converges with the outlet stream of the proglacial lake of the Roseg glacier just above a braided floodplain. At the downstream end of this floodplain, the braided channels converge and the river flows through a narrow valley before entering the Bernina river, a tributary to the Inn river (Fig. 1C).

Over the last 25 years, between the first sampling in 1997 and the last sampling in 2022, the uppermost site located just below the glacier terminus has shifted 1.1 km upward. In the same period, the Tschierva glacier has lost 2.1 km^2^ in surface area, which is over 30% of the mass it had in 1997. Figure 1A shows the recession of the glacier snout for the Tschierva and Roseg glaciers over a 125-year time span, portraying the dramatic decrease in mass in the last century as well as the entire glacial coverage of the Roseg valley in the years of sampling. The longitudinal study was based on previous studies in 1997 (Burgherr and Ward 2001) and 2008 (Finn et al. 2010) using the same sites from each study as well as new upper sites in relation to ongoing glacial recession. In this study, sites were selected to be as close as possible to sites of the previous studies as well as two additional sites in the new channel emerging with glacial recession since 2008. We used the distance from the Bernina confluence as a continuous variable for comparing sites among the three collection dates. The length of the study river was split into four zones for longitudinal comparisons (Fig. 1C), depending on the specific analyses described below. The distance (thalweg line) to the downstream confluence of the Roseg River and Bernina River regarding the delineation between zones A/B is 10.2 km, zones B/C is 9.0 km, and zones C/D is 7.0 km. The three studies included similar sites, except for 2008 where the lower valley was not sampled. The coordinates of the study sites for each sampling year are found in Table S1. All sites in all years were sampled in summer (August–September).

### Lake network study

**Fig. 2:**
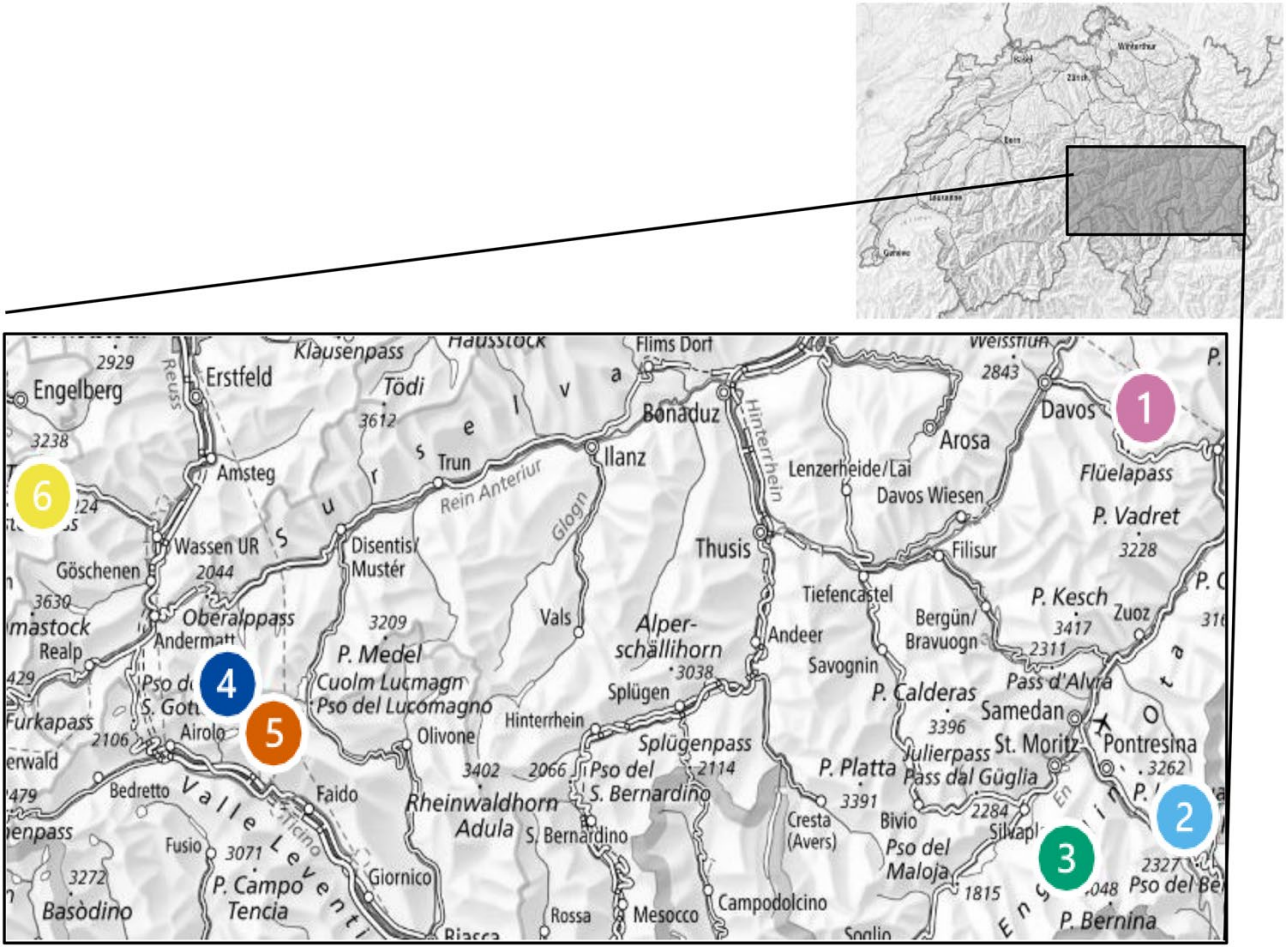
Map of the site locations in the lake network study; 1. Joeri, 2. Pouz Minor, 3. Roseg, 4. Scuro, 5. Stabbio, and 6. Stein

For this study, six alpine lakes sampled in late August 1998/99 were revisited during September 2022 (Fig. 2). Four lakes were situated in the northern Alps: lake Stein in canton Bern south of Susten Pass in the Uri Alps, lake Joeri near Davos, Switzerland in canton Grison, and lakes Pouz Minor and Roseg near Pontresina, Switzerland in canton Grison. The other two lakes, Lago Scuro and Lago di Stabbio, lie in the southern Alps in the Quinto region in canton Ticino. Lakes Stein and Roseg are kryal systems, being fed by the Stein and Roseg glaciers, respectively. Conversely, the other four lakes are rhithral systems, primarily supplied by rainwater and snowmelt in spring. Data from 1998 was used for lakes Stein, Joeri, Pouz Minor, and Roseg (Hieber et al. 2005), and from 1999 for Lago Scuro and Lago di Stabbio (Donath and Robinson 2001). For all lakes except Lago di Stabbio, sampling was conducted at the lake inlet, lake outlet, and a downstream site between 200 and 300 m below the outlet. For Lago di Stabbio, only the lake outlet was sampled due to the absence of an inlet and inaccessibility of a downstream site (the stream flowed over a cliff face 60 m from the outlet).

## Methods

In the longitudinal study, historical flow data were analyzed to understand how the flow regime was affected by glacial recession. The gauging station was located just above the confluence with the Bernina river and has been operated by the Swiss National Hydrological and Geological Survey since 1955. For this study, we used monthly average discharge between years, and monthly as well as yearly average discharge over the whole 25-year sampling period. The flow data were matched with air temperature records (Swiss Federal Office of Meteorology and Climatology; MeteoSwiss) for possible correlations because increases in discharge are often related to a rise in air temperature in kryal systems (Uehlinger et al. 2003). Here, we used mean and maximum air temperature measured 5 cm above the grass layer at the meteostation in Samedan, Switzerland about 10 km distance from the hydrologic gauging station and at a similar altitude.

In both studies, water physico-chemistry was assessed using a 0.5-L sample collected from each site prior to sampling of macroinvertebrates. Sample analysis in the laboratory included pH, alkalinity, total hardness, chloride, nitrate, sulfate, phosphate, total phosphorus (TP), dissolved organic carbon (DOC), and total organic carbon (TOC) following Tockner et al. (1997). In addition, we field measured temperature and electrical conductivity (WTW LF 330; Wissenschaftliche-Technische Werkstätten (WTW), Germany), pH (WTW pH 330i), dissolved oxygen (WTW Oxi 330; WTW Germany), and turbidity (Cosmos; Züllig, Switzerland) as nephelometric turbidity units (NTU) of the surface waters.

For all sites in both studies, near-bed velocity (MiniAir 2; Schiltknecht Messtechnik AG, Switzerland) and water depth were measured at 50 random spots along each site. For determining mean substrate size at a site, 50 random stones were measured (b-axis, cm). Elevation and coordinates for each site were determined using a handheld GPS-tracker (eTrax 22x, Garmin Switzerland). Channel width was measured at three equidistant locations along each site. Lastly, the Pfankuch index of river stability was calculated at each site. This index incorporates 15 variables that can be determined in the field, providing an estimate of the general stability of a given site (Pfankuch 1975; Collier 1992).

Sample collection at all sites in both studies followed the same procedures as the earlier studies. Sites comprised around 30 m along the main channel. For macroinvertebrates, three Hess samples (area = 0.043 m^2^, 250 µm mesh) at three different locations at each site were collected and preserved in 70% ethanol. A mesh size of 100 μm was used in 1997 for Hess samples in the longitudinal study, restraining some data analyses (see below). All samples were processed in the laboratory using a stereomicroscope. Macroinvertebrates were identified after Tachet (2010), counted, and stored in 90% ethanol for archiving. On the basis of the previous studies, macroinvertebrates were identified to genus for most taxa and sub-family for the Chironomidae. As with previous years, only taxa that made up more than 1% in any one sample were considered for comparing among years.

For organic resources, we measured periphyton on stones and benthic organic matter from each Hess sample; both quantified as ash-free dry mass (AFDM). For periphyton, we randomly collected five submerged stones at each site and stored them frozen at − 20°C until processed. In the laboratory, the surface of each stone was scrubbed, and the area measured. The scraped residue was filtered, dried at 60 °C, weighed, combusted at 500 °C for 3 h, and reweighed. The difference in weights was an estimate of periphyton AFDM on a stone. For determining benthic organic matter, the remaining material of each Hess sample after removing macroinvertebrates was used. This material was processed as AFDM following the same protocol used for periphyton.

### Data analysis

#### Longitudinal stream study

All data analyses were performed using R Statistical Software (v 4.2.3; R Core Team 2023).

All environmental measures were log(1 + *x*) transformed (except pH) and then used in the Principal Component Analyses (PCA) to compare temporal and spatial differences among sites. Three different PCAs were conducted because of novel technological developments and different laboratory protocols used for some measures over the 25-year study period. One PCA was run that included all sites and years using a relatively few common parameters among years, another PCA included years 2008 and 2022 using additional measures common to both years, and the last PCA used only the 2022 data with all measures collected in that year.

Macroinvertebrate data were calculated as percentages of total abundance per site to account for different mesh sizes of the Hess sampler between 1997 (100 µm) and 2008/2022 (250 µm) that compromised comparison of total abundance data. Ecological indices of Simpson index, Shannon index and Taxa richness were calculated using the R package vegan (v2.6-4) and acted as response variables in regression models using temperature and site distance from the Bernina river as predictors. We used a Nonmetric Multidimensional Scaling (NMDS) analysis to examine the spatial and temporal differences in community assemblages using asin(sqrt) transformed percent abundance data with the Adonis package in R. We then tested for differences in diversity indices and the first NMDS axis among the four zones along the river using analysis of variance (ANOVA) followed by Tukey’s post-hoc test for significant results (Zar 1984). We used an analysis of covariance (ANCOVA) to test for Taxa richness differences among years at the proglacial sites with distance as predictor and sampling year as a covariate. ANCOVA also was used to investigate the relationship between biodiversity indices and temperature as a predictor and year as a factor variable. Generalized additive models (GAMs) were used to account for the non-linearity in the diversity along the whole length of the stream (Cauvy-Fraunié et al. 2014b) using distance as a continuous predictor (with a smoothing term) and year as a factor variable. Here, the response variables were Shannon index, Simpson index, and Taxa richness. Because water temperature is closely related to the dispersal of the macroinvertebrates in glacial streams (Milner et al. 2001), another GAM was calculated with temperature as the predictor variable. GAMs were built using the R package *mgcv* (Wood 2017).

#### Lake network study

Some data-use differences existed in the two earlier studies, constraining some comparisons in the lake network study. In Hieber et al. (2005), abundance data for macroinvertebrates were available as well as the same environmental data collected in this study except substrate size. In Donath and Robinson (2001), only presence/absence data for macroinvertebrates were available, whereas abundance data were absent. Their study also included most of the environmental data used in this study except for near-bed velocity and substrate size. A log(*x* + 1) transformation was applied to the environmental and water chemistry data (except pH) prior to data analysis using a principal component analysis (PCA). A Quade test was used for comparing principal components on environmental factors between historical and present data as well as for comparison of lake inlets, lake outlets, and downstream sites (Quade 1979). For macroinvertebrate community data, non-metric multidimensional scaling (NMDS) was conducted, with subsequent permutational multivariate analysis of variance (PERMANOVA) using distance matrices to test for group differences (Clarke 1993; McArdle and Anderson 2001). Prior to analysis, the macroinvertebrate abundance data were log(1 + *x*) transformed. Macroinvertebrate diversity indices, including Taxa richness, Shannon index, and Simpson index were calculated using the vegan package in R Studio. A Scheirer-Ray-Hare test was used for comparison of data over time, including Shannon and Simpson indices, Taxa richness and selected physico-chemical measures. Pairwise Wilcoxon Rank Sum Tests were used for comparisons of diversity indices and Taxa richness among lake inlets, lake outlets and downstream sites.

## Results

### Longitudinal study: patterns in physico-chemistry

**Fig. 3:**
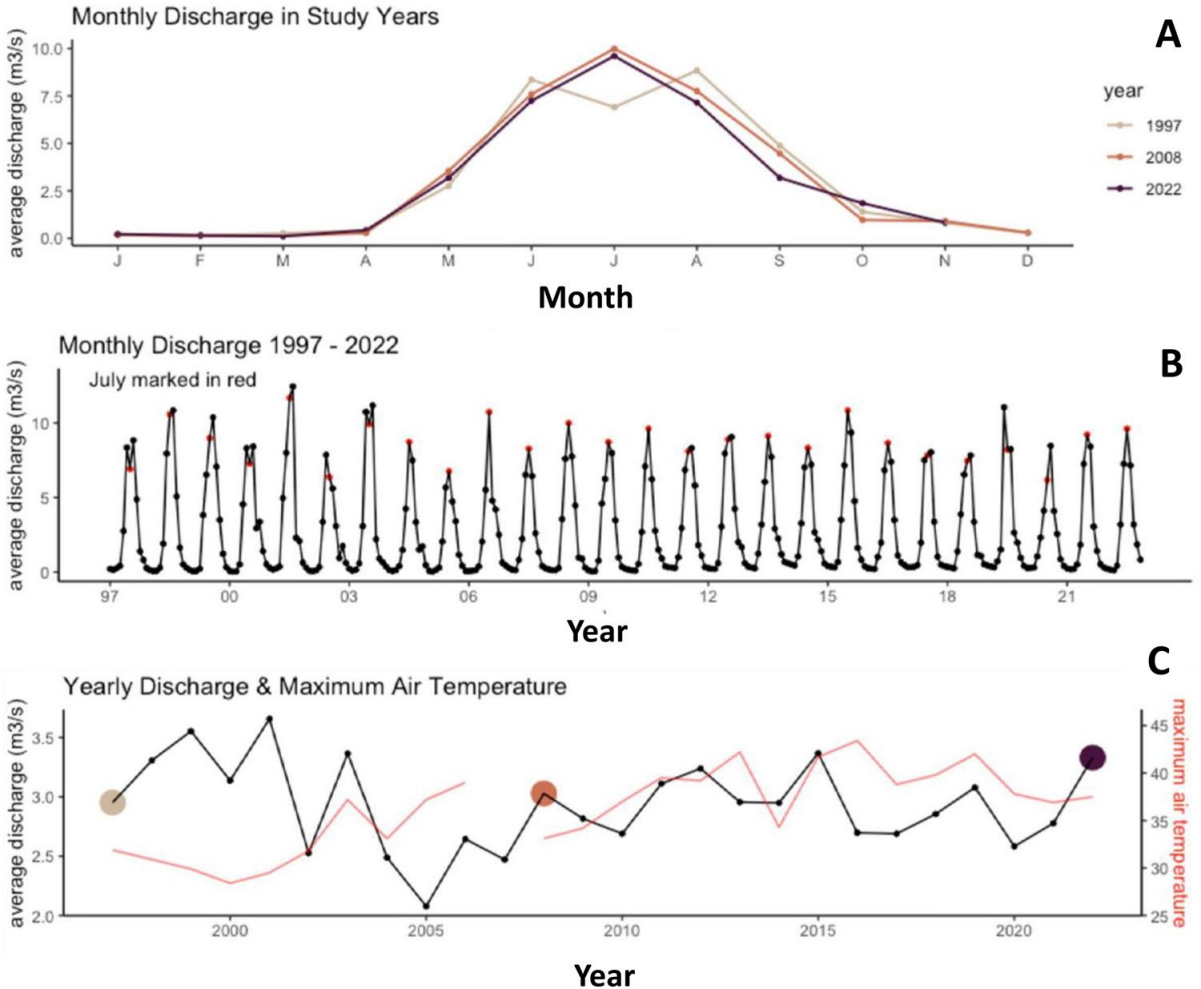
**A** Average monthly discharge (m^3^/s) in the Roseg River for the different study years. **B** Average monthly discharge (m^3^/s) across years of the study (1997 until 2022) showing the peak flow each year that represents the annual differences in weather, e.g., dry years versus wet years. **C** Annual discharge (m^3^/s) over the study period (1997–2022) along with values of maximum air temperatures (°C) for the same period

Annual discharge patterns showed no visible differences among study years (Fig. 3A). The monthly flow averages among study years were similar as well as the timing of peak flows in summer and low flows in winter. The flow pulse remained steady over the 25 years of study, and flow maxima occurred with no clear change in timing (Fig. 3B). The maximum annual air temperature showed an increase over time and with coherent patterns with annual average discharge after 2008 (Fig. 3C). In years before 2008, the maximum temperature was lower and did not appear to reflect differences in discharge.

**Fig. 4:**
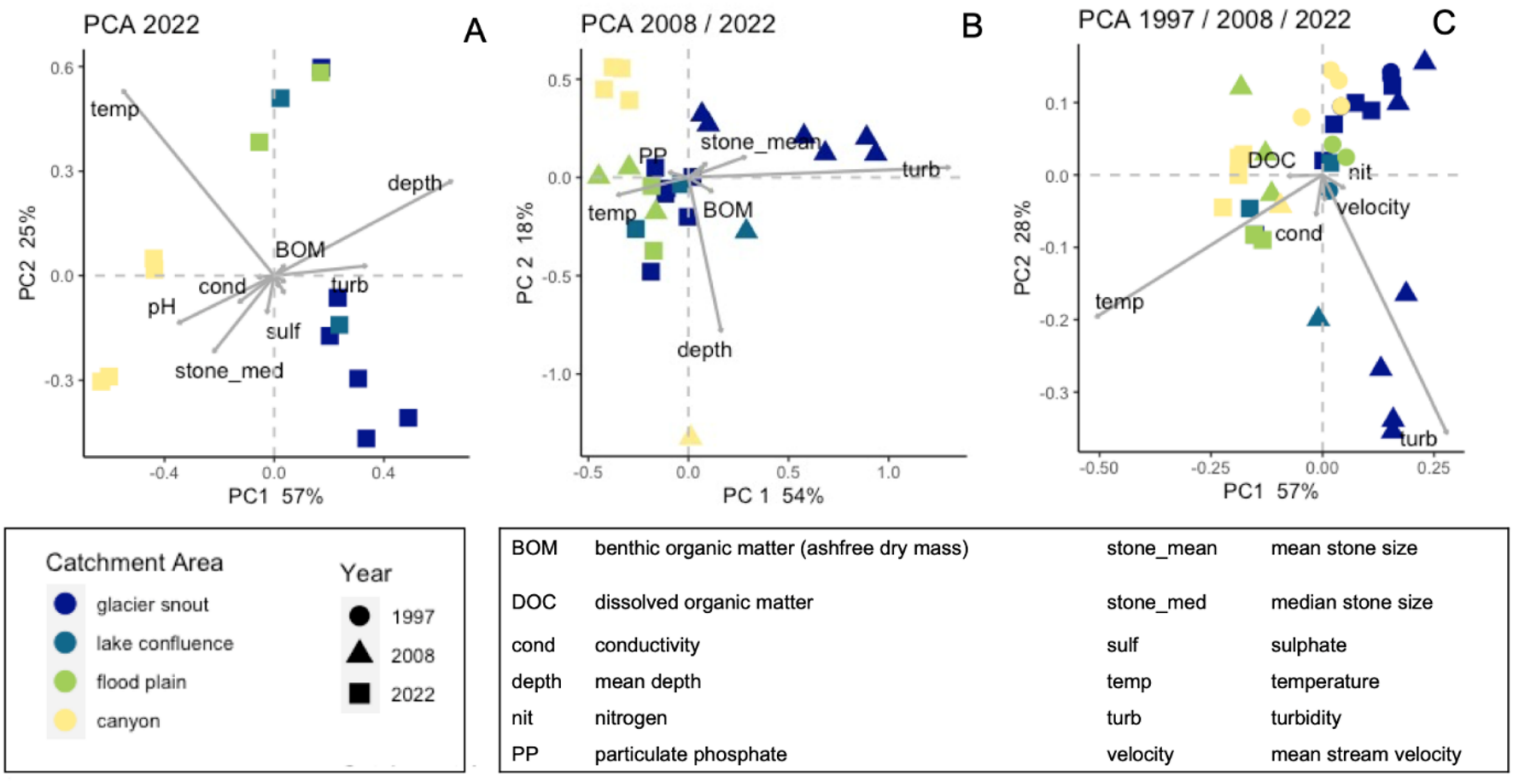
Principal components analysis (PCA) results based on physico-chemical measures comparing sample sites in **A** study year 2022, **B** study years 2008/2022, and **C** study years 1997/2008/2022. Measures used in each comparison differed due to changes in analytical protocols and differences among measures collected each study year. The colors of symbols represent the different zones as labeled in Fig. 1, where glacial snout is zone A, lake confluence is zone B, flood plain is zone C, and canyon is zone D. Only variables with loadings > 0.70 are shown on the plots

In all three PCAs, temperature and turbidity were loaded highly on the first two axes (Fig. 4). In all three PCAs, temperature and turbidity separated the cooler, more turbid proglacial sites from the warmer, less turbid lower sites. The PCA for data from 2022 indicated depth, temperature, pH, and turbidity were the main contributors to axis-1, whereas temperature, depth and median substrate size best explained axis-2 (Fig. 4A). These two axes explained 82% of the variation in the data. In the PCA using data from 2008 and 2022 (Fig. 4B), turbidity, temperature and mean substrate size best explained the variation along axis-1, while depth was loaded high on axis-2 and separated the lower valley sites from the upper sites. These two axes accounted for 72% of the variation in the data in this PCA. In the PCA using data from all three years (Fig. 4C), axis-1 was best described by turbidity and axis-2 by temperature, together explaining 85% of the variation in the data.

### Longitudinal study: patterns in macroinvertebrates

Overall, average Taxa richness increased to 23 in 2022 from 17 in 1997 and 21 in 2008. Nineteen taxa recorded in the earlier studies were present in 2022, except for the family Tipulidae (Diptera) and, in 2008, Capniidae (Plecoptera). The subfamily Diamesinae represented up to 70% of the abundances in samples in 2022. *Siphonoperla* (Plecoptera: Chloroperlidae) and *Rhabdiopteryx* (Plecoptera: Taenioperygidae) were first documented 2008 and recorded also in 2022. The genus *Ecdyonurus* (Ephemeroptera: Heptageniidae), which was first reported in 2008 with low abundances in the lower floodplain, was found abundant at all sites below the lake confluence in 2022.

**Fig. 5:**
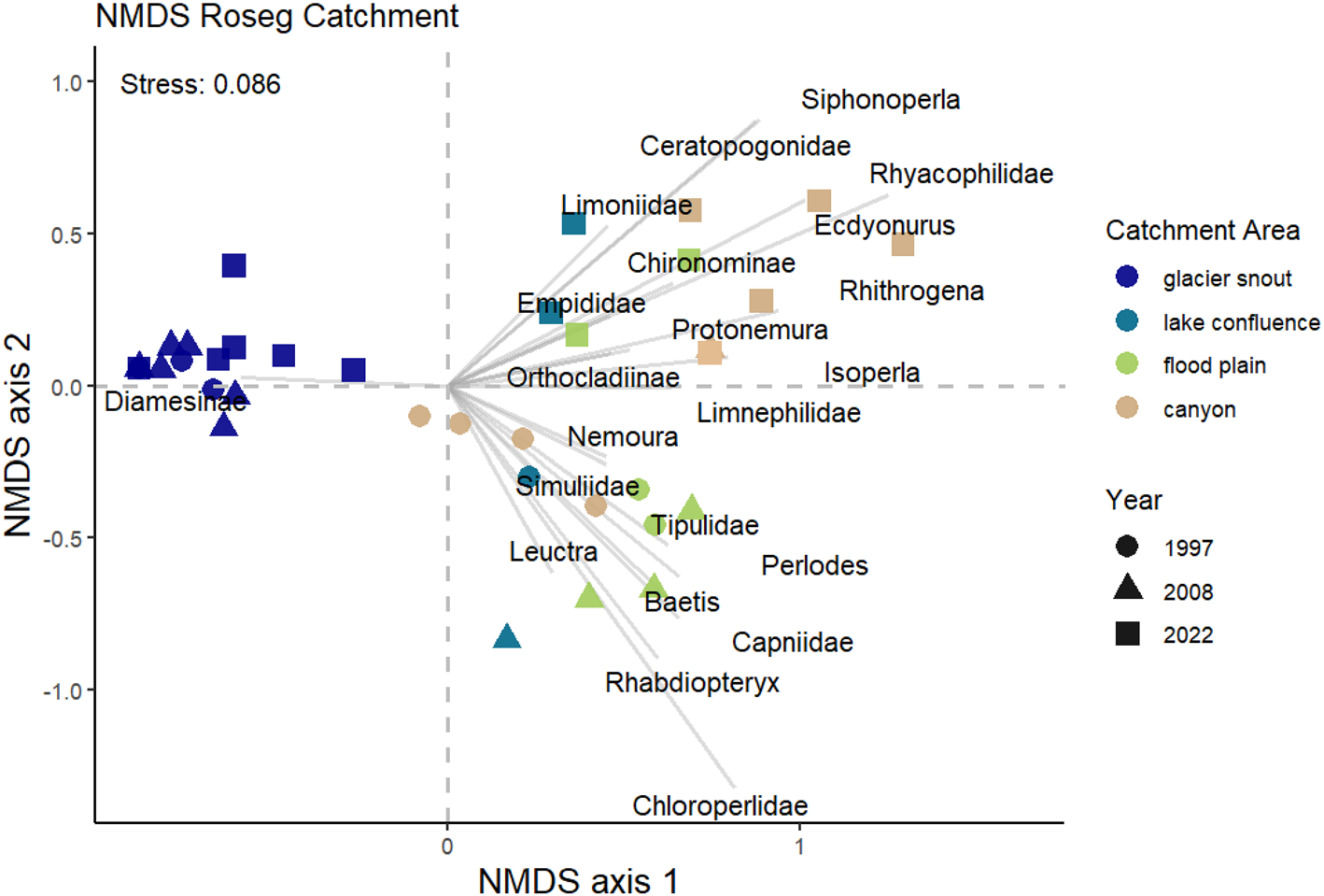
NMDS results (stress = 0.09) based on macroinvertebrate assemblages collected at the different sites in the different study years (1997, 2008, 2022). Catchment area indicates the different zones as delineated in Fig. 1. No canyon collections were made in 2008

The NMDS results clearly separated the glacial snout sites from the other sites by the presence of Diamesinae on the left side of axis-1 versus the presence of other taxa on the right side of axis-1 (Fig. 5). For the lower sites, there was a detectable gradient among the different sampling years along axis-2 with 2022 sites located higher along this axis than the same sites in 1997 and 2008. For example, the lower valley (canyon) sites were clearly separated in 1997 from 2022, as well as for flood plain and lake confluence sites in 2022 relative to 1997 and 2008 (Adonis; *R*^2^ = 0.58, *p* < 0.00; Fig. 5). For the lower sites, the difference between years was greater than the difference between zones (Adonis; *R*^2^ = 0.14, *p* < 0.032).

**Fig. 6:**
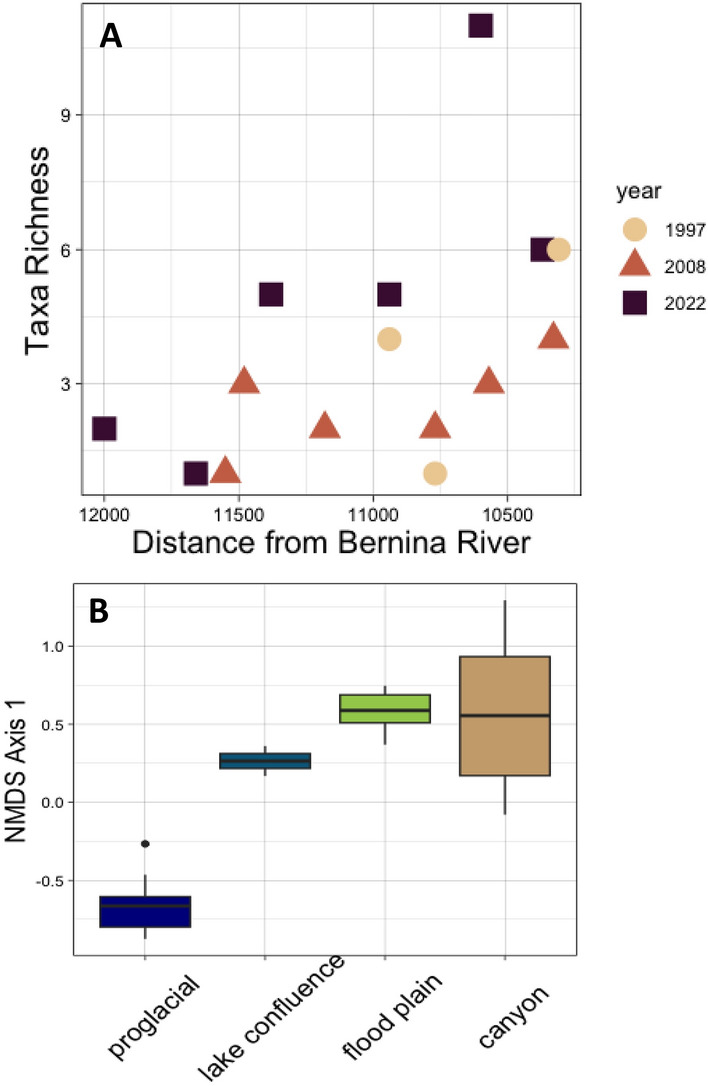
**A** Macroinvertebrate taxa richness among study years with distance from the Bernina river confluence. **B** Box plots (mean, 95% confidence limits and standard deviation) of the NMDS Axis-1 scores of the macroinvertebrate assemblage data (see methods) separated for each zone along the Roseg River shown in Fig. 1. Here, glacial snout is zone A, lake confluence is zone B, floodplain is zone C, and canyon is zone D

Taxa richness significantly decreased with distance from the Bernina river (*F* = 6.73; *p* = 0.03), and taxa richness increased at individual sites over the study period (Fig. 6A). The regression models also showed a linear upward shift of taxa into newly emerging proglacial sites. The upper most site in each year typically had the lowest abundances and were dominated by the subfamily Diamesinae. There was a clear difference in NMDS axis-1 scores between the zones (*F* = 61.65; *p* < 0.001) with the glacial snout zone (A) differing from the other zones (Tukey’s, *p* < 0.001) (Fig. 6B).

**Fig. 7:**
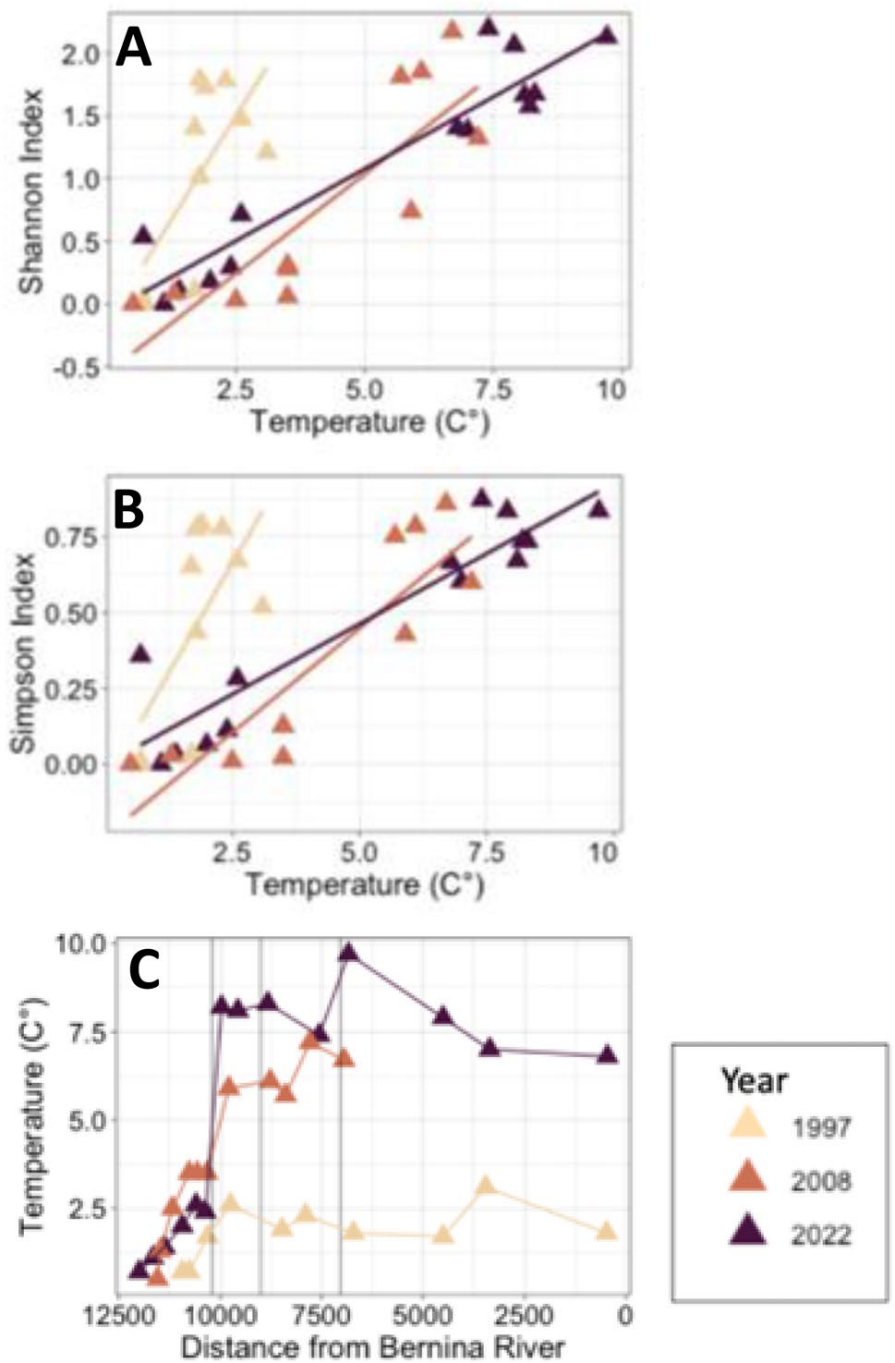
Relation of **A** Shannon Index and **B** Simpson Index with water temperature among study years. **C** Measures of water temperature with distance from the Bernina river confluence for the different study years. Here, the vertical lines indicate the border between the different zones as depicted in Fig. 1

The three diversity indices were strongly correlated with distance from the Bernina river (all, *p* < 0.001) and all three indices showed a peak in diversity in the floodplain zone (Supplement Fig. 1). Further, the diversity indices showed significant correlations with temperature (all, *p* < 0.001) and with different slopes for each year of study (Fig. 7A–C). The peak in diversity occurred at much lower temperatures in 1997 (around 2.5 °C) than in 2008 and 2022 (around 6–8 °C). Mean temperatures increased from sites in the glacial snout to sites in the confluence zone owing to the influx of water from the lake by 2.3 °C in 1997 (SD = 0.4), 6.2 °C in 2008 (SD = 0.7), and 8.0 °C in 2022 (SD = 0.4) (Fig. 7C).

### Lake network study: patterns in physico-chemistry

**Fig. 8:**
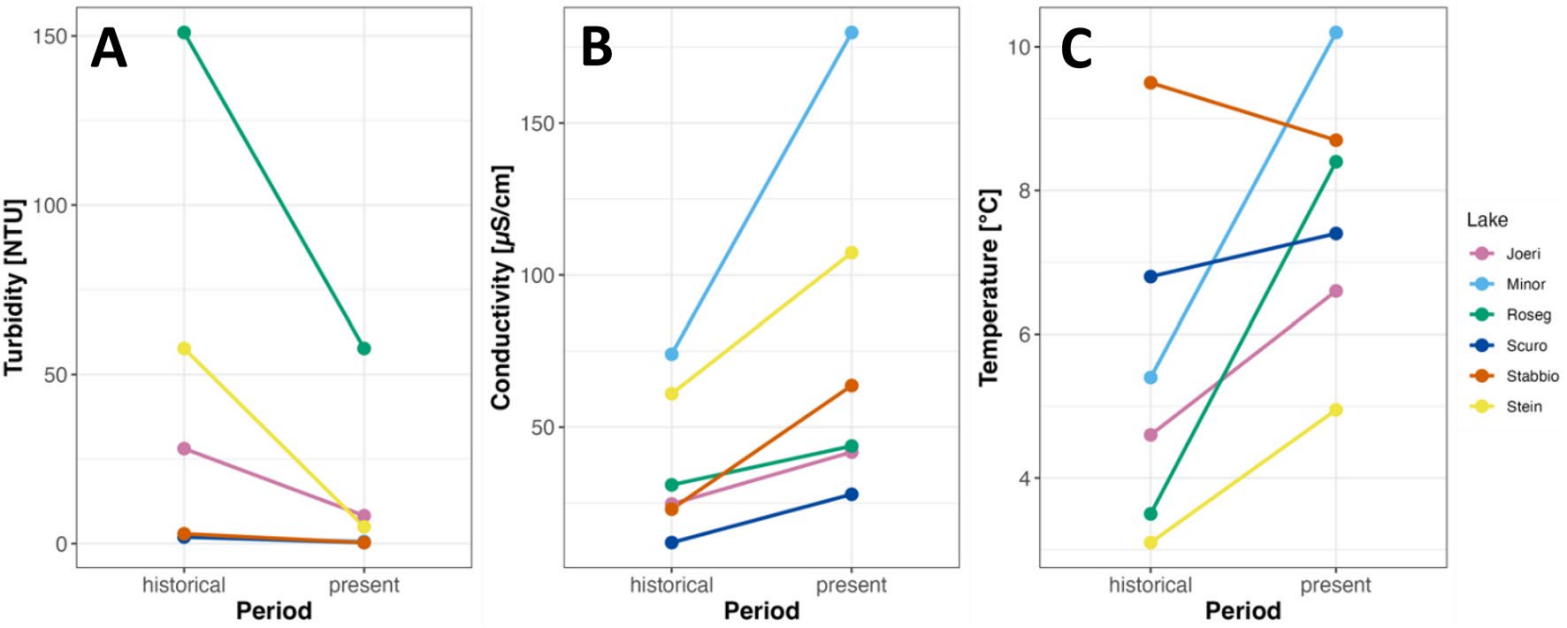
Changes in surface water **A** turbidity (NTUs), **B** conductivity (μS/cm) and **C** temperature (°C) between 1998/99 (historical) and 2022 (present) for all 6 study lakes

In all lake outlets, an increase in conductivity was observed between the two time periods, especially in lakes Pouz Minor, Stein and Stabbio (Fig. 8). Notably, lake outlets in the northern Alps (Joeri, Pouz Minor, Roseg, Stein) showed substantial decreases in turbidity and increases in water temperature between the two time periods, whereas lake outlets in the southern Alps (Scuro, Stabbio) showed similar turbidities and temperatures between the two time periods (Fig. 8).

**Fig. 9:**
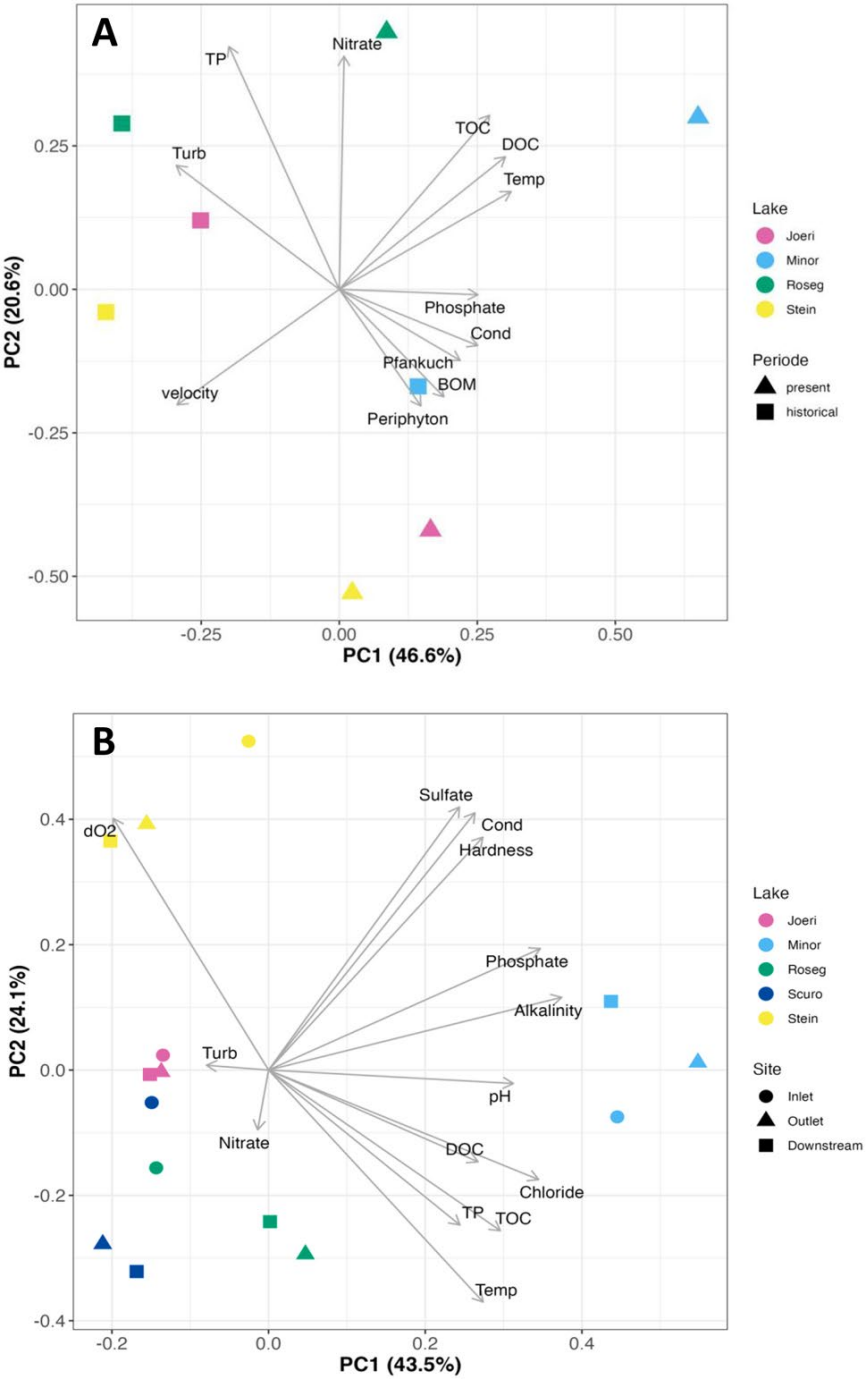
Principal components analysis (PCA) results on physico-chemical measures collected in **A** 1998/99 (historical) and 2022 (present) at four different lake outlets (Joeri, Minor, Roseg, Stein), and **B** at lake inlets, outlets, and downstream sites sampled in 2022 at lakes Joeri, Pouz Minor, Roseg, Scuro, and Stein. The explained variance of the axes in both plots are labeled. Notations: *TP* total phosphorus, *TOC* total organic carbon, *DOC* dissolved organic carbon, *Turb* turbidity, *Temp* temperature, *Cond* conductivity, *BOM* benthic organic matter, and *dO*_*2*_ dissolved oxygen. Only variables with loadings > 0.70 are shown

The PCA biplot of physico-chemical data for northern lake outlets (Joeri, Pouz Minor, Roseg, Stein) showed a notable increase along axis-1 for all lake outlets (Quade test; *F* = 15, *p* = 0.031) (Fig. 9A). Axis-1 explained 46.6% of variation in the data and was best explained by temperature, dissolved organic carbon (DOC), turbidity, velocity, total organic carbon (TOC), soluble reactive phosphate and conductivity. Axis-2 explained 20.6% of variation in the data and was best explained by total phosphorus (TP), nitrate, total organic carbon (TOC), benthic organic matter (BOM), dissolved organic carbon (DOC) and periphyton biomass. There was no significant difference along axis-2 among lake outlets (Quade test; *F* = 1.94, *p* = 0.30) or time (Quade test; *F* = 0.46, *p* = 0.55). Kryal lake outlets (Roseg, Stein) showed lower values than rhithral lake outlets (Joeri, Pouz Minor) on axis-1 for both periods (Fig. 9A).

Specifically, the lake outlet in Joeri increased in temperature by 2°C between the two periods (from 4.6 to 6.6 °C) and an increase in conductivity from 24.7 to 41.8 μS/cm. The outlet of Pouz Minor increased in temperature from 5.4 to 10.2 °C, and conductivity increased from 74 to 180 μS/cm. Roseg outlet increased in water temperature from 3.5 to 8.4 °C between the two periods as well as a major decrease in turbidity from 151 to 57.5 NTUs and total phosphorus from 52 to 21.7. Here, periphyton biomass increased by 13.2 mg/m^2^ between periods. Lake Scuro’s outlet increased in conductivity from 12 to 27.9 μS/cm. At Stabbio outlet, periphyton biomass decreased from 45.8 to 24.1 mg/m^2^. Lastly, the outlet of lake Stein increased in water temperature between the two periods from 2.3 to 3.9 °C and conductivity increased from 61 to 107.3 μS/cm. The Stein outlet decreased in turbidity from 57.5 to 5.0 NTUs, but periphyton biomass increased from 3.3 to 24.6 mg/m^2^.

The PCA biplot of physico-chemical data in 2022 comparing lake inlets, outlets and downstream sites explained 43.5% of variation along axis-1 and 24.1% of variation along axis-2 (Fig. 9B). PCA axis-1 was best explained by alkalinity, soluble reactive phosphate, chloride, pH, total organic carbon (TOC), hardness, and dissolved organic carbon (DOC). PCA axis-2 was best explained by sulfate, conductivity, dissolved oxygen, and temperature. There were differences noted among lakes along axis-1 (Quade test; *F* = 4.77, *p* = 0.029) as well as axis-2 (Quade test; *F* = 5.36, *p* = 0.021). However, no significant differences were found among inlet, outlet, or downstream sites for each lake (Quade test; PC1: *F* = 0.932, *p* = 0.43; PC2: *F* = 0.661, *p* = 0.54).

There were some lake specific differences in physico-chemistry observed among lake sites in 2022. In Joeri, the downstream site had much higher turbidity (373 NTUs) and benthic organic matter (15.3 g/m^2^) compared with the inlet and outlet (mean NTUs: 7.5, BOM: 5.9 g/m^2^). The inlet, on the other hand, had the highest total phosphorus (7.6 versus mean 3.6 μg/L) and the outlet had the highest periphyton biomass (26.1 versus mean 10.4 mg/m^2^). In Pouz Minor, the inlet had higher turbidity (7.5 versus mean 0.79 NTUs), and total phosphorus (33.1 versus mean 12.1 μg/L), but less conductivity (119.1 versus mean 179.8 μS/cm), sulfate (33.4 versus mean 60.7 mg/L) compared with the outlet and downstream site. The inlet of Roseg had lower conductivity (27.4 versus mean 43.8 μS/cm), temperature (5.4 versus mean 8.3 °C), turbidity (14.0 versus mean 61.8 NTUs), total phosphorus (11.6 versus mean 43.8 μg/L), and sulfate (6.1 versus mean 9.2 mg/L) compared with the outlet and downstream site. The inlet of Scuro had higher values in conductivity (35.2 versus mean 15.7 μS/cm), nitrate (0.33 versus mean 0.11 mg/L) and sulfate (3.7 versus mean 1.1 mg/L) but lower benthic organic matter (4.2 versus mean 7.0 g/m^2^) and periphyton biomass (7.2 versus mean 14.1 mg/m^2^) compared with the outlet and downstream site. At Stein, the inlet had the highest values of conductivity (201.4 versus mean 107.4 μS/cm), turbidity (14.9 versus mean 7.4 NTUs), sulfate (80.1 versus mean 40.1 mg/L), total phosphorus (7.7 versus mean 3.9 μg/L), and benthic organic matter (2.2 versus mean 1.4 g/m^2^).

### Lake network study: patterns in macroinvertebrates

Overall, 60 taxa were collected and identified from all lake outlet sites. Dipterans were most abundant, contributing 40% to the total number of macroinvertebrates. The Chironomidae represented 36% of the total number of individuals and Orthocladiinae was the most abundant chironomid at 43%, followed by Diamesinae 22%, Tanypodinae 15% and Tanytarsini 14%. The pediciids represented 4% and simuliids 2.7% of the Diptera. Mayflies (Ephemeroptera) made up 10% of total abundances with *Beatis* sp. representing 51%, *Rhithrogena* sp. 28%, and *Ecdyonurus* sp. 10%. Stoneflies (Plecoptera) contributed 6% of the total abundances of macroinvertebrates with the most frequent being *Leuctra* sp. at 24% and *Nemoura* sp. 17%. Other recorded stoneflies included *Siphonoperla* sp., *Capnia* sp., *Protonemura* sp., *Isoperla* sp., *Dictyogenus* sp., *Perlodes* sp., *Rhabdiopteryx* sp., and *Chloroperla* sp. The caddiesflies (Trichoptera) were the least abundant taxa, making up only 2% of total macroinvertebrates. Drusinae at 44%, Limnephilinae 29%, and Rhyacophilidae 14% were common Trichoptera. *Hydra* sp. contributed 28% and Ostracoda 9% of the total abundances of macroinvertebrates recorded in samples.

**Fig. 10:**
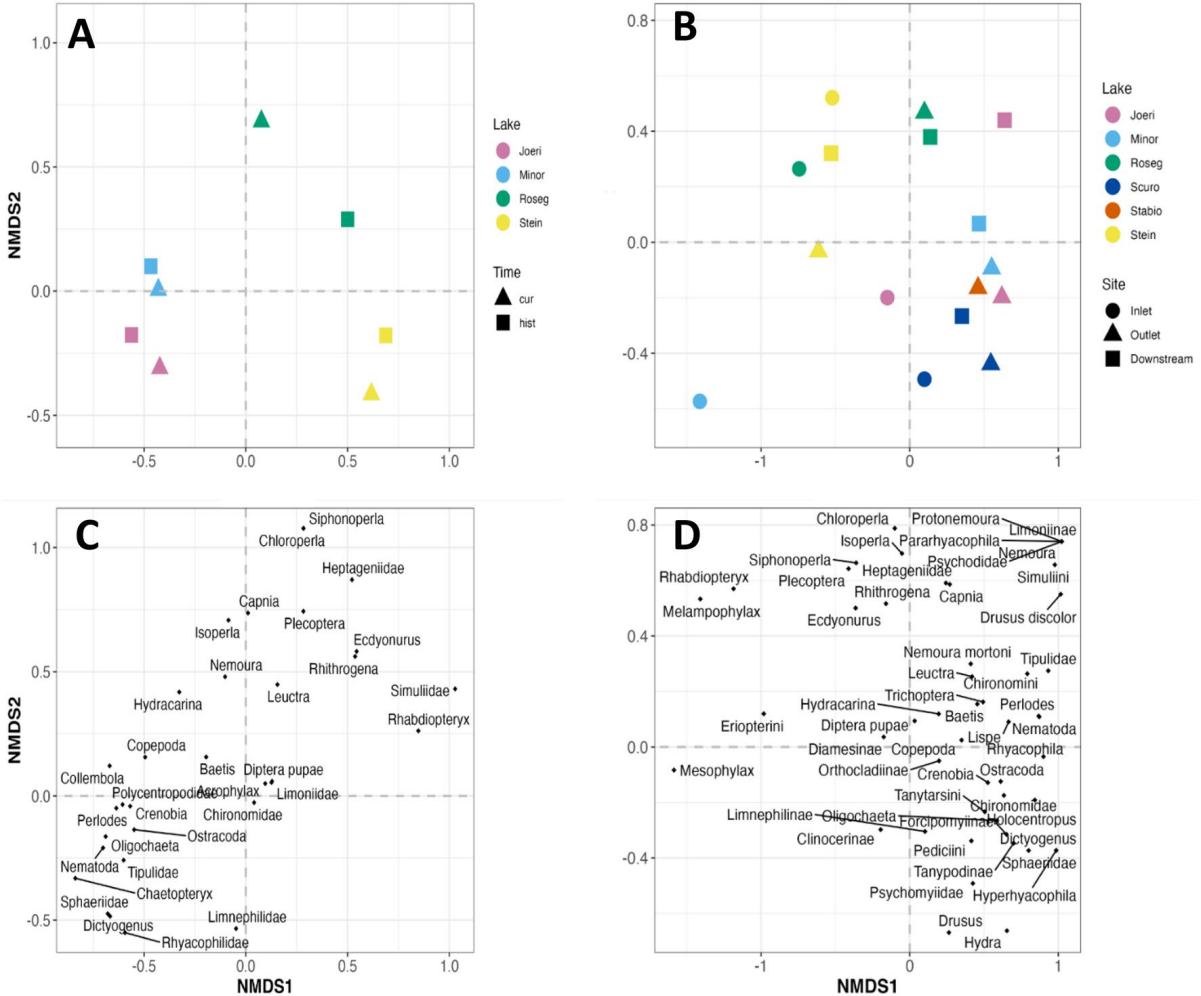
Non-metric multidimensional scaling (NMDS) results using macroinvertebrates collected in **A** 1998/99 (historical) and 2022 (present) at four different lake outlets (Joeri, Pouz Minor, Roseg, Stein), and **B** at lake inlets, outlets, and downstream sites sampled in 2022 at lakes Joeri, Pouz Minor, Roseg, Scuro, Stabbio and Stein. Only the outlet was sampled at Stabbio as no inlet stream was present and no access was available for a downstream site

Nonmetric multidimensional scaling (NMDS) analysis of the abundance data showed no overall shifts in species composition at lakes between study periods (Fig. 10A). PERMANOVA results indicated no significant changes over time in the outlets (*F* = 1.55, *p* = 0.19), but notable differences were found among lakes (*F* = 3.72, *p* = 0.007) as well as between rhithral and kryal outlets (*F* = 4.42, *p* = 0.014). Kryal outlets were separated from rhithral outlets along axis-1 of the NMDS in the present as well as historical data. Roseg showed the largest shift in community composition between periods, whereas Stein, Joeri and Minor had similar values between periods.

The NMDS comparing inlet, outlet and downstream sites showed differences among lakes and sites at lakes (Fig. 10B). The PERMANOVA indicated differences among inlet, outlet, and downstream sites (*F* = 2.13, *p* = 0.024), differences between kryal and rhithral lakes (*F* = 3.55, *p* = 0.005), as well as differences between northern and southern lakes (*F* = 2.43, *p* = 0.012). There was a large difference in community composition between inlets and outlets and downstream sites at lakes Pouz Minor and Roseg. The downstream site of Joeri was separated from the inlet and outlet sites. Kryal lakes clustered together with negative values along NMDS axis-1 and southern-Alpine lakes were on the right site of the NMDS with higher values on axis-2.

### Lake network study: patterns in diversity indices

**Fig. 11:**
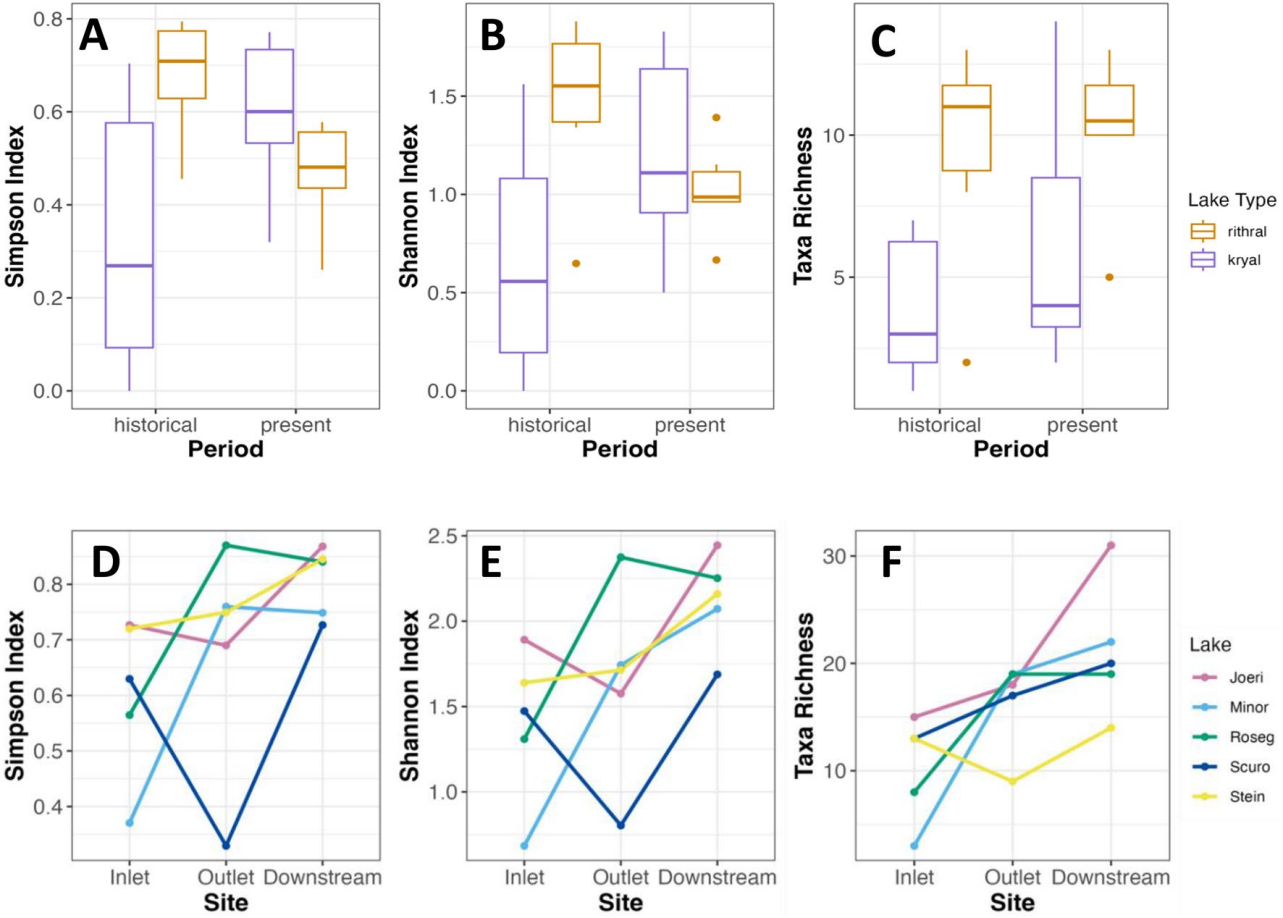
Changes in macroinvertebrate assemblages as **A** Simpson Index, **B** Shannon Index, and **C** Taxa Richness for outlet samples grouped by kryal and rhithral lake types for 1998/99 (historical) and 2022 (present) data as well as **D** Simpson Index, **E** Shannon Index, and **F** Taxa Richness between lake inlets, outlets, and downstream sites for lakes Joeri, Pouz Minor, Roseg, Scuro, and Stein in 2022

Contrasting patterns were observed when comparing diversity differences in kryal and rhithral lake outlets over time (Fig. 11); e.g., Simpson index (*H* = 7.36, *p* = 0.006) and Shannon index (*H* = 4.32, *p* = 0.037) changed over time depending on lake type. Taxa richness was notably different between lake types in both periods (*H* = 7.28, *p* = 0.007), but showed no significant difference over time. An overall increase was found in Simpson index (*p* = 0.048), Shannon index (*p* = 0.048) and Taxa richness (*p* < 0.001) between lake inlets and downstream sites. Simpson index (*p* = 0.002) and Shannon index (*p* < 0.001) differed between outlet and downstream sites, whereas Taxa richness was similar (*p* > 0.05). Inlets had a lower Taxa richness than outlets (*p* = 0.027) and downstream sites (*p* < 0.0001). Lastly, Taxa richness was lower in kryal than rhithral systems (*p* = 0.024).

### Lake specific patterns in diversity

At Joeri, there was a decrease in Simpson and Shannon indices between the two study periods. There was a large increase in the abundance of Diptera (chironomids), whereas other groups did not change in abundance. New taxa at Joeri included Tipulidae, Rhyacophilidae, and Leuctridae. The lake inlet as well as the downstream site had higher Shannon and Simpson values compared with the lake outlet. Taxa richness, on the other hand, was higher at the downstream site (31 taxa) than at the outlet (18 taxa) and inlet (15 taxa) sites.

At Pouz Minor, the Shannon and Simpson indices decreased between study periods. There was a large increase in Diptera (chironomids) but a decrease in Ephemeroptera (*Baetis* sp.) abundance. Limoniidae were present in 1998 but not found in 2022, and Tipulidae were new to the system. The inlet had lower Simpson and Shannon indices as well as Taxa richness (3 taxa) compared with the outlet (19 taxa) and downstream (22 taxa) sites.

The Roseg outlet showed an increase in Shannon and Simpson indices as well as Taxa richness between study periods. There was a large increase in Plecoptera (*Capnia* and *Leuctra* spp.) abundance. New taxa included *Ecdyonurus* sp., *Chloroperla* sp*., Isoperla* sp. and *Siphonoperla* sp. Simuliidae were absent in recent samples. Roseg inlet had lower Shannon index, Simpson index and Taxa richness at the inlet (8 taxa) than outlet and downstream sites (both 19 taxa).

At Scuro, *Hydra* sp. was novel in 2022 with a large abundance, whereas *Baetis* sp., *Dyctiogenus* sp., *Drusus* sp*. Protonemoura* sp., Simuliidae and *Crenobia* sp. were absent. Other new taxa were Copepoda, Empididae, *Nemoura* sp. and *Philopotamus* sp. The Shannon and Simpson indices were lower at the outlet compared with the inlet and downstream sites. Taxa richness was lower in the inlet (13 taxa) than outlet (17 taxa) and downstream (20 taxa) sites.

Taxa richness increased from 17 to 21 taxa at Stabbio. New taxa in 2022 included Ceratopogonidae, Clinocerinae, Forcypomyiinae, Limnephilidae, *Nemoura* sp., *Philopotamus* sp., whereas Tipulidae. Limoniidae, *Leuctra* sp., Lepidoptera and *Agabus* were absent in recent samples.

At Stein, the Shannon and Simpson indices increased between study periods, as well as Taxa richness (from three to seven taxa). The abundance of Diptera (chironomids) decreased drastically, and no Limoniidae were found in 2022. Newly recorded taxa included *Ecdyonurus* sp., Limnephilidae, and Taeniopterygidae. In general, macroinvertebrate abundances were low. The downstream site had greater Shannon and Simpson indices compared with the inlet and outlet sites. Taxa richness was lowest in the lake outlet (9 taxa) compared with the lake inlet (13 taxa) and downstream (14 taxa) sites.

## Discussion

This study summarized the long-term patterns of alpine macroinvertebrates (1) longitudinally along the glacial stream in Val Roseg in response to glacial recession and (2) among six different lake networks, including various physical–chemical measures recorded at the different sample dates for both projects. The former documented the upstream colonization of newly emerged streams as well as upward trends in earlier upper sites by various macroinvertebrates, whereas the latter compared temporal patterns among lake inlets, lake outlets and downstream sites over 24/25 years. Both studies revealed major response patterns by alpine waters and macroinvertebrates under ongoing glacial recession and landscape transformation due to environmental change.

### Longitudinal stream study

Discharge recorded at the gauging station at Pontresina showed little variation among years over the 25-year period with no visible pattern of either an increase or decrease. Even when data since 1954 were included, the mean annual discharge showed no pattern in variation and the variation among years seemed related to maximum air temperatures in respective years. Rapid glacial mass loss is initially correlated with an increase in discharge as rising air temperatures accelerate ice melt (Milner et al. 2017). With increased glacial run off, water temperature drops as the thermal capacity is enhanced (Van Vliet et al. 2013), while turbidity increases. Water temperature rises as discharge declines only after the peak of mass ice loss. Data collected over the 25-year span in Val Roseg suggest that these glacial systems are already past the rapid mass loss stage. This result is consistent with the findings from the upper Aare and Rhône catchments in Switzerland where historical flow trends show the peak in melt-water runoff was around the mid-twentieth century (Collins 2008). In Val Roseg, there is still enough permanent ice to ensure a maximum discharge around July that potentially buffers stream intermittency (Fleming 2005; Robinson et al. 2016b), although the complete loss of permanent ice in the valley is expected in the late twenty first century (Huss et al. 2010).

This loss in ice seems to be true for many of the small glaciers in Europe, although larger ice masses also will face further loss (Zemp et al. 2006). Questions remain on how it will affect freshwater ecosystems and their inhabitants. Several studies on different organisms in glacial meltwater streams reveal predictions for the future of these habitats. In Milner et al.’s study (2008) on a deglaciated stream in Alaska, the sub-family Diamesinae went extinct after water temperatures went above 7.5 °C. This response would indicate a loss of this taxon simultaneously with the loss of glaciers in Val Roseg. However, other studies showed that the loss of glacial mass does not have to equal the loss of taxa, as rhithral systems (Muhlfeld et al. 2020) and rock glacier waters (Hotaling et al. 2017) could provide refugial conditions for cold-adapted species. Some species also could find refuge in spring-fed (krenal) streams (Giersch et al. 2017), which show a broader diversity compared with kryal streams (Kownacki 1991). Further, Sertić Perić et al. (2021) examined food webs and food resources in alpine streams and found that the loss of ice mass could lead to narrower feeding niches as major changes in hydrology could limit primary production, altering basal resources for consumers. On the other hand, warmer water temperatures could bring benefits for other taxa that live below the extreme habitats of the glacier snout and limited in their physiology to adapt to colder temperatures below their optimum (Madsen et al. 2015). Data from the Roseg shows that the abundance of Diamesinae in the proglacial reach has decreased from around 98% in 1998 and 2008 to 91.6% in 2022, suggesting that a population shift in this cold-water habitat is likely occurring.

Another important variable reflecting a glacial water influence was turbidity. Previous studies have shown how turbidity is higher near the glacier snout and decreases downstream (Milner et al. 2017; Cauvy-Fraunié et al. 2014a). In the 2008 data, turbidity at upper sites was dramatically higher (up to 15× higher) than in 1997 or 2022. This result could be due to a landslide or local storm that mobilized debris from the glacial moraine into the stream. For instance, turbidity values in 2008 stabilized again below the lake confluence, most likely through dilution with less turbid lake outlet water. In the other study years, turbidity values showed the anticipated decrease away from the glacier. Interestingly, the high turbidity values in 2008 did not have a large influence on macroinvertebrate diversity or abundance. As the surrounding moraine is rather unstable, and prevalent for landslides, the organisms in these streams may be adapted and resilient to such episodic events.

There was a clear upward shift by macroinvertebrates at newly-emerged proglacial sites, being colonized and dominated by the well-adapted Diamesinae (see Finn et al. 2010). Further, the earlier upper sites in 1997 and 2008 also were colonized by novel taxa in 2022 that previously were not found above the confluence with the lake outlet stream. This upward shift by taxa, analogous to the terrestrial realm, can threaten endemic alpine species adapted to the harsh environmental conditions of proglacial streams (Wilkes et al. 2023). In addition, the loss of permanent ice with the vertical retreat of glaciers up mountains causes stream slopes to be steeper, increasing channel instability that can negatively affect macroinvertebrate abundances (Milner et al. 2001). With the reduced glacial influence, the habitat for Diamesinae (and other endemic alpine taxa) will gradually decrease and other taxa such as Orthocladiinae, an alpine chironomid adapted to cold-water systems at lower elevations, may replace them (Robinson et al. 2016a). Further, only Leuctridae represented stoneflies in the respective lowest proglacial site in the former studies, while *Protonemoura*, *Nemoura* and *Isoperla* were present in 2022 as well as the mayfly *Baetis alpinus*. Similar colonization patterns have been observed in other glacial streams in Switzerland (see Robinson et al. 2014).

Below the confluence with the proglacial lake outlet stream, glacial waters appeared buffered by various environmental factors, e.g., the local influence of the braided flood plain (Ilg and Castella 2006), that affected biotic assemblages and possibly dispersal dynamics (e.g., Shama et al. 2011). For instance, braided streams offer heterogeneous habitats that are generally positively correlated with biodiversity (Huston 1994; Benda et al. 2004; Rahbek et al. 2019). This effect was supported by the decrease in diversity indices below the braided floodplain where the waters merge into a single channel in the lower valley, leading to the observed hump-shape pattern in diversity as shown in other alpine studies (Jacobsen and Dangles 2012; Cauvy-Fraunié et al. 2014b).

Alpine lakes can act as source or sink habitats (Arp and Baker 2007) and be heat traps (Sakai et al. 2000), increasing water temperatures in outlet streams. Here, the dramatic difference between mean outlet temperatures in 1997 and 2008/2022 can be explained partially by the separation of the proglacial lake from the glacier in the latter years. For instance, glacial attachment to proglacial lakes has a cooling effect on lake water temperature and thus outlet streams (Carrivick and Tweed 2013). The mean lake outlet temperature was 3.5 °C in 1997 (Hieber et al. 2002), whereas in 2022 the newly emerged inlet stream had a mean temperature of 5.4 °C and the outlet was 8.4 °C. The influence of warmer outlet water below the confluence is substantial and clearly altered the temperature profile. Nevertheless, the temperature increase is not the only variable influencing macroinvertebrate distributions. Of interest here is the rise in diversity below the confluence observed in all study years, even in 1997 when the water temperature increase below the confluence was substantially lower than in later years. We suggest that channel stability likely increased below the confluence as the system shifted to a more open braided floodplain as mentioned above.

The results of this study confirm the expected upward shift in macroinvertebrates with glacial recession. The resulting increase in alpha-diversity also brings a change in stream food webs. In proglacial sites, basal resources stem mainly from the gold alga *Hydrurus foetidus* during the autumn and winter, as well as diatoms (Uehlinger et al. 2010). With less turbidity and higher stream temperature, different autotrophs may colonize with the reduction in glacial flow (Rott et al. 2006; Besemer et al. 2007). This change in resources also could support novel secondary consumers and thus broaden food webs. Although we found periphyton levels remained the same from 2008 to 2022, novel macroinvertebrates were moving upstream with glacial recession. The relation between periphyton assemblages and macroinvertebrates in proglacial streams under glacial recession is still an open question. It is therefore important to further monitor the systems, especially indicator organisms such as macroinvertebrates (Khamis et al. 2014) and diatoms (Peszek et al. 2022), to improve our predictions and adapt protective actions if needed.

### Lake network study

Overall, six alpine lake networks initially sampled in 1998 were revisited 2022 to investigate how macroinvertebrate assemblages have evolved over two decades in response to changing environmental conditions. Alpine lakes, located in high-altitude regions between the tree line and permanent snow line, are sensitive indicators of environmental change due to their unique characteristics (Bretschko 1995; Füreder et al. 2006). Turbidity, a parameter reflecting water clarity, exhibited a consistent decrease across all the study systems over the study period, and is consistent with the observations of reduced suspended sediment concentrations reported in previous research resulting from climate-induced glacier shrinkage (Chang et al. 2001; Huss et al. 2008; Bellard et al. 2012). Conversely, electrical conductivity, a measure of ion concentration, showed a notable increase in lake outlets and is in line with global projections due to increased weathering and ion leaching from catchments as temperatures rise (Houghton et al. 2001). Further, the northern-Alpine lakes (Pouz Minor, Joeri, Roseg, and Stein) experienced a substantial increase in water temperature over the study period reflecting the trend of above-average warming in the region (BAFU 2020; Michel et al. 2021). The notable changes in physico-chemistry indicate potential shifts in the physical–chemical character of alpine lake outlets as alluded to in Robinson et al. (2022). Comparing the physico-chemical parameters among lake inlets, outlets, and downstream sites also revealed some intriguing patterns. For instance, turbidity and conductivity in Roseg and Stein decreased from inlet to outlet, being attributed to the settling of suspended particles as water flows through the lake (Catalan et al. 2006). Further, an increase in temperature from inlet to outlet were observed at some lakes like Stein. Downstream sites, however, had a more variable temperature range, reflecting local topography and solar exposure in shaping stream water temperatures (Catalan et al. 2006).

More specifically, the multivariate analysis of physico-chemistry data revealed distinct patterns of increase in some variables among the studied lakes. Axis-1 of the PCA included variables such as alkalinity, soluble reactive phosphate, chloride, pH, total organic carbon (TOC), hardness, and dissolved organic carbon (DOC), indicating the study lakes shared a commonality in respect to regional factors such as geology and climate (Füreder et al. 2001; Ward 1994). Axis-2, on the other hand, was explained by sulfate, conductivity, oxygen, and temperature, and emphasized the local differences among lakes that influenced surface water physico-chemistry of outlet streams (Füreder et al. 2001; Hannah et al. 2007). The analysis of habitat characteristics also demonstrated that local factors influenced instream habitats of the lake outlets, including water depth and width, flow velocity, substrate size, and organic resources such as periphyton biomass and benthic organic matter. These habitat differences were attributed to variations in catchment characteristics, local topography, and respective glacier contributions (Füreder et al. 2001; Hannah et al. 2007).

Macroinvertebrate assemblages at lake outlets provided insight into how these ecosystems have changed in response to shifting environmental conditions. Notably, lake outlets exhibited higher diversity compared with respective lake inlets, suggesting that lake outlets are transition zones that support a diverse macroinvertebrate assemblage in response to enhanced resource availability and habitat heterogeneity of outlets (Luoto and Nevalainen 2013). Additionally, downstream sites generally exhibited higher taxa richness than lake inlets and lake outlets, suggesting that downstream areas may act as reservoirs for other taxa, potentially receiving inputs from both lake outlet and downstream sources (also see Robinson and Minshall 1990; Harding 1992). Further, the multivariate analysis (NMDS) of species composition revealed that kryal systems tended to cluster together and were distinct from rhithral systems. Kryal systems generally showed lower taxa richness than rhithral systems, being attributed to the distinct environmental conditions and hydrological characteristics of these two stream types (Hieber et al. 2005). For example, the higher taxa richness in rhithral streams may be owing to warmer temperatures, lower turbidity and higher periphyton biomass (Hieber et al. 2005). This distinction underscores the importance of hydrological dynamics and water source in shaping macroinvertebrate assemblages in alpine lake networks (Hieber et al. 2005).

A comparison of responses among lake outlets provided insight into the varying impacts of climate change and environmental alterations on macroinvertebrates in alpine ecosystems. Joeri outlet experienced a substantial increase in taxa richness and significant shifts in community composition, likely attributed to the warming trend observed in the northern-Alpine lakes (Michel et al. 2021). As temperatures rise, it is likely that new taxa, particularly those adapted to lower-altitude environments, colonize alpine lake outlets. This underscores the potential for alpine ecosystems to serve as refugia for species seeking cooler habitats as temperatures increase in lower-altitude environments (Catalan et al. 2006). In contrast, Pouz Minor outlet had a decrease in diversity, linked to changes in water chemistry, including increased conductivity and alterations in nutrients (Füreder et al. 2001; Houghton et al. 2001). Further, Roseg outlet exhibited an increase in both taxa richness and diversity due to an increase in Plecoptera abundance (i.e., *Leuctra* sp.) and new taxa such as *Capnia* sp. (Catalan et al. 2006). Additionally, the absence of certain taxa, including *Baetis* sp. and Simuliidae, is noteworthy and warrants further investigation. Both taxa are common inhabitants of alpine streams in general, thus their absence may be related to a biotic interaction such as competition or predation. At Stein outlet, the absence of Limoniidae in the recent data indicates a shift in the Diptera community (Lods-Crozet et al. 2001).

Lake inlets generally exhibited lower diversity compared with outlets, suggesting inlets of alpine lakes may represent more challenging habitats for macroinvertebrates owing to factors such as colder temperatures, higher turbidity, and limited resource availability (Luoto and Nevalainen 2013). Downstream sites, on the other hand, displayed higher diversity compared with inlets and outlets, consistent with the idea that downstream areas often act as transition zones, receiving inputs of organic matter and nutrients from both the lake outlet and upstream catchment, thereby supporting a more diverse array of species (Harding 1992; Hieber et al. 2005). While some lakes exhibited an increase in richness from inlet to outlet, others displayed the opposite trend. Pouz Minor, for instance, experienced a notable increase in richness from inlet to outlet, likely driven by changes in water temperature and habitat availability (Catalan et al. 2006). In contrast, Stein showed a decrease in richness from inlet to outlet, potentially linked to changes in water chemistry influenced by the lake (Füreder et al. 2001). In some cases, such as Scuro, the inlet, outlet, and downstream sites cluster together in multivariate space, suggesting minimal differences in community structure and habitat properties. Conversely, in Joeri, these sites exhibited distinct spread, indicating substantial differences in community composition and habitat properties. Variations in community composition are attributed differences in habitat structure, water chemistry, and the presence of specific taxa (Füreder et al. 2001; Hieber et al. 2005). In cases where sites cluster together, stream segments may share more similar ecological conditions, potentially owing to the influence of factors such as glacial meltwater contributions or catchment characteristics (Ward 1994; Catalan et al. 2006).

The observed variations in physio-chemical parameters among lake sites emphasize the need to consider the entire lake-catchment continuum when assessing environmental changes in alpine waters. Climate-driven alterations in water chemistry, turbidity, and temperature can have profound implications for macroinvertebrate communities and overall ecosystem integrity (Füreder et al. 2001; Michel et al. 2021). Additionally, the biotic responses observed across lake sites highlight the importance of differentiating between inlet, outlet, and downstream habitats when assessing biodiversity patterns. Outlets and downstream sites often emerged as biodiversity hotspots, supporting a diverse array of species due to the combined influence of inflowing nutrients and the presence of diverse mesohabitats (Robinson and Minshall 1990; Hieber et al. 2005). Decreasing turbidity at these sites reflects improved water clarity, likely due reduced glacial influence caused by glacier shrinkage and changes in sediment dynamics. Conversely, increasing conductivity and temperature signal shifts in water chemistry and thermal regimes, potentially impacting the composition and distribution of aquatic life. These habitat changes transcend the boundaries of individual lake sites, emphasizing the importance of considering the interconnectedness of the entire fluvial network.

The higher biodiversity in outlets compared with inlets suggests that lake outlets may represent transitional zones supporting more diverse macroinvertebrate assemblages. However, downstream sites often emerged as biodiversity hotspots, emphasizing their role as hubs of ecological diversity. Community composition patterns among lakes and habitats highlighted the uniqueness of each lake’s ecological structure. In a broader context, these findings shed light on the resilience and vulnerability of high-altitude ecosystems in the face of climate change. Alpine lakes, with their sensitivity to environmental shifts, serve as valuable indicators of broader ecological transformations. The observed variations emphasize the need for adaptive conservation strategies that consider the heterogeneity of these systems and their responses to ongoing environmental changes. As we move forward, it is important to continue monitoring alpine lake ecosystems, considering the entirety of their hydrological and ecological gradients. Comprehensive assessments are essential for preserving these pristine environments and the invaluable biodiversity they harbor, even as they confront the challenges posed by a changing world.

## Data Availability

All data can be requested from the corresponding author.
